# Comparative analysis of signal accuracy of three SpO_2_ monitors during motion and low perfusion conditions

**DOI:** 10.1007/s10877-023-01029-x

**Published:** 2023-06-02

**Authors:** Karen K Giuliano, Robert N Bilkovski, John Beard, Sakari Lamminmäki

**Affiliations:** 1 Elaine Marieb Center for Nursing and Engineering Innovation, University of Massachusetts Amherst, Institute of Applied Life Sciences and Elaine Marieb College of Nursing, 240 Thatcher Road, Amherst, MA 01003 USA; 2 RNB Ventures Consulting Inc., 12191 W. Linebaugh Avenue, Unit 687, Tampa, FL 33626 USA; 3 Patient Care Solutions, GE HealthCare, 8200 W. Tower Ave, 53223 Milwaukee, WI USA; 4 Patient Care Solutions, GE HealthCare, Teollisuuskatu 29, 00510, Helsinki, Finland

**Keywords:** Pulse oximetry, Motion-artifact, SpO_2_, Low perfusion

## Abstract

To compare pulse oximetry performance during simulated conditions of motion and low perfusion in three commercially available devices: GE HealthCare CARESCAPE ONE TruSignal SpO_2_ Parameter, Masimo RADICAL-7 and Medtronic Nellcor PM1000N. After IRB approval, 28 healthy adult volunteers were randomly assigned to the motion group (N = 14) or low perfusion (N = 14) group. Pulse oximeters were placed on the test and control hands using random assignment of digits 2–5. Each subject served as their own control through the series of repeated pair-wise measurements. Reference co-oximetry oxyhemoglobin (SaO_2_) measurements from the radial artery were also obtained in the motion group. SpO_2_ readings were compared between the test and control hands in both groups and to SaO_2_ measurements in the motion group. Accuracy was assessed through testing of accuracy root-mean squared (ARMS) and mean bias. In the simulated motion test group the overall Accuracy Root Mean Square (ARMS) versus SaO_2_ was 1.88 (GE), 1.79 (Masimo) and 2.40 (Nellcor), with overall mean bias of − 0.21 (Masimo), 0.45 (GE), and 0.78 (Nellcor). In the motion hand, ARMS versus SaO_2_ was 2.45 (GE), 3.19 (Masimo) and 4.15 (Nellcor), with overall mean bias of − 0.75 (Masimo), − 0.01 (GE), and 0.04 (Nellcor). In the low perfusion test group, ARMS versus the control hand SpO_2_ for low PI was 3.24 (GE), 3.48 (Nellcor) and 4.76 (Masimo), with overall bias measurements of − 0.53 (Nellcor), 0.96 (GE) and 1.76 (Masimo). Experimental results for all tested devices met pulse oximetry regulatory and testing standards requirements. Overall, SpO_2_ device performance across the three devices in this study was similar under both motion and low perfusion conditions. SpO_2_ measurement accuracy degraded for all three devices during motion as compared to non-motion. Accuracy also degraded during normal to low, very low, or ultra low perfusion and was more pronounced compared to the changes observed during simulated motion. While some statistically significant differences in individual measurements were found, the clinical relevance of these differences requires further study.

## Introduction

In 1974 in Japan, Takuo Aoyagi and Akio Yamanishi independently filed patents which became the foundational science for current pulse oximetry technologies [1]. Since then, pulse oximetry has become a widely recognized standard of care for numerous clinical applications where the monitoring of oxygen saturation, or oxyhemoglobin, is required to optimize outcomes. Pulse oximetry is likely the most commonly used medical device for both inpatient and outpatient care [2] and is associated with more rapid detection and treatment of respiratory compromise, particularly in the perioperative setting [3, 4].

Pulse oximetry (SpO_2_) technology requires an arterial pulse signal, light emitting diodes, and a photo detector. In clinical settings, an oximeter is used to transmit light with red and infrared wavelengths most commonly through the tissues of the finger or ear. The tissue absorbs much of the emitted light, while the remainder passes through the tissue to be measured by a light-sensitive photodiode. As oxygen saturation increases, the more infrared light is absorbed by oxyhemoglobin and the more red light is transmitted through. Conversely, as oxygen saturation decreases, the more red light is absorbed by deoxyhemoglobin and the more infrared light is transmitted through. The measured ratios of red to infrared light transmission by the photodiode allow for the calculation of the percentage fraction of oxygenated hemoglobin, resulting in a displayed clinical SpO_2_ reading [5–7]. While SpO_2_ values are noninvasive estimates of oxyhemoglobin levels, SaO_2_ levels provide direct measurements of oxyhemoglobin levels as collected through arterial blood samples. SaO_2_ measurements are considered the gold standard for oxyhemoglobin assessment [8].

Pulse oximeter accuracy may be impacted by multiple factors including, but not limited to, low perfusion, motion, skin pigmentation, dyshemoglobinemias, anemia, dyes, nail polish, and ambient light [8]. Low perfusion states, such as sepsis or cardiogenic shock, may decrease SpO_2_ accuracy because pulse oximetry requires a sufficient arterial pulse signal which is often decreased during these conditions. Patient movement, such as that associated with shivering or delirium, creates artifacts which interfere with SpO_2_ measurement and impact measurement accuracy. These inaccuracies place patients at risk for delayed or missed recognition of hypoxemia. Precise SpO_2_ readings are especially important for the safe care of critically ill patients.

Pulse oximetry inaccuracy related to skin pigmentation is currently being reevaluated by the United States Food and Drug Administration (FDA), who held a Medical Devices Advisory Committee meeting on pulse oximetry in November of 2022 [8]. This inaccuracy may have notable implications for the treatment of patients with varying skin pigmentation [6, 9, 10].

Pulse oximetry technology has improved over time to reduce measurement errors, including those caused by motion and low perfusion. The use of these newer algorithms has been shown to improve clinical performance by reducing both data dropout and false alarms [11, 12]. However, even with these improvements, studies performed in laboratory settings using either a high-fidelity simulator or healthy volunteers and simulated conditions demonstrate that motion and low perfusion continue to present challenges for measurement accuracy [13, 14]. While differences in the measurement accuracy of various pulse oximeters have been reported, no specific type or brand of pulse oximeter has been found to be superior overall [14, 15]. The combination of low perfusion and increased levels of skin pigmentation may pose additional challenges to the accuracy of pulse oximetry measurements [16].

Variations in pulse oximetry accuracy may be caused by hardware, software and algorithms, wireless connectivity, and other design elements which have been introduced to maximize signal quality and reliability across a variety of challenging clinical conditions. Thus, the purpose of this study was to evaluate and compare the accuracy of three currently available pulse oximeters: (GE HealthCare CARESCAPE ONE TruSignal SpO_2_ Parameter, Masimo RADICAL-7, Medtronic Nellcor PM1000N SpO_2_) under simulated conditions of motion and low perfusion in a group of healthy volunteers.

## Methods

### Design

This was a prospective, open-labeled comparative evaluation of three commercially available pulse oximeters: (GE HealthCare CARESCAPE ONE TruSignal SpO_2_ Parameter, Masimo RADICAL-7, Medtronic Nellcor PM1000N SpO_2_) under conditions of motion and low perfusion across four phases of oxygenation. This study was approved by the University of California, San Francisco Committee on Human Research (San Francisco, California) and written informed consent was obtained from all participants. The study design was aligned with ISO 80601-2-61:2017 and FDA Guidance for Pulse Oximeter Pre-Market Notification Submissions [17].

### Study participants

Twenty-eight healthy adult (≥ 18 to < 50 years) volunteer subjects were enrolled. Inclusion criteria were good general health, non-smokers, and normal hemoglobin (≥ 10 g/dL). Exclusion criteria were obesity, serious systemic illness, diabetes, cardiovascular disease, pulmonary disease, Raynaud’s disease, clotting disorders, and pregnant or lactating females. Subject enrollment was designed to meet FDA guidance requirements of a minimum of two darkly pigmented subjects or 15% of the total pool, whichever is larger [17]. Skin pigmentation was categorized by the 6-level Fitzpatrick Scale [18, 19].

Half of the subjects (N = 14) were randomly assigned to the motion protocol and the other half (N = 14) were randomly assigned to the low perfusion protocol. A minimum threshold of measurement pairs was included in accordance with ISO 80601-2-61:2017. The 14-subject sample size for each protocol meets FDA requirements [FDA] for the study and is consistent with other published analyses of similar technologies [2, 13]. The study was not powered to undertake subgroup analysis.

### Protocol

Subjects in both motion and low perfusion groups had three pulse oximeters placed on both a test hand (motion or low perfusion) and control hand (non-motion or normal perfusion). Pulse oximeters were randomly assigned to digits 2 to 5 on both test and control hands to mitigate for order bias.

Subjects were administered air–nitrogen–carbon dioxide mixtures with a voluntarily increased minute ventilation, with carbon dioxide added as needed to maintain normocapnia. The test administrator adjusted the inspired air–nitrogen–carbon dioxide mixture breath-by-breath to achieve a series of stable SaO_2_ plateaus at desired saturation levels. The stable saturation plateau was maintained for at least 60 s with SpO_2_ fluctuating by less than 2–3%. This method has been used in previous studies [13] and typically requires a period of time for the oxygen saturation to stabilize. The controlled desaturation study procedure followed the guidelines of pulse oximetry standard ISO 80601-2-61:2017: Annex EE.2 PROCEDURE for invasive laboratory testing on healthy adult volunteers (motion group) and Annex EE.3 PROCEDURE for non-invasive laboratory testing on healthy adult volunteers (low perfusion group). ISO 80601-2-61:2017:Annex EE.2 proposes to have ≥ 30 s plateau before blood sample.

### Data collection

#### Motion testing

In the motion group, each subject had two control blood samples taken at the beginning of each experiment, while breathing room air. For each subject, desaturation was repeated six times to reach a low SpO_2_ plateau (SpO_2_ target 85–90%) with a period of high SpO_2_ plateau (approximately 92–100%) between each round. At each SpO_2_ plateau, a blood sample was taken and used to perform pair-wise comparisons of the test hand and control hand SpO_2_ measurements against the CO-oximeter SaO_2_.

Motion was induced palm down using a clenching technique, pressing and rubbing motion (CPR), palm up with twitching/clenching (T/C), and a tapping motion (Tap). Motion conditions were generated by the test subjects with variable intensity and frequency. Oximeters were recorded continuously to collect SpO_2_ readings across each saturation plateau. SpO_2_ readings were compared between the test and control hands and to simultaneous SaO_2_ measurements to assess accuracy. The motion methodology was adapted from a study by Tobin et al. [20] characterizing the motion artifact types in hospitalized patients. Subject generated motion was also used more recently in another study by Louie [13]. Compared to machine generated motion, this study method has more variability and is more clinically relevant as simulation of patient movement. To ensure that the motion conditions are approximately equal across the tested devices, the test subjects were observed during the testing and instructed to keep motion between sensors equal. To randomize the possible intensity differences between fingers, the sensors were rotated between fingers after three of six desaturation cycles for each subject.

Arterial blood was sampled (in total N = 248 GE, N = 250 Nellcor and Masimo) at each saturation plateau to obtain SaO_2_ values. Data are grouped into SaO_2_ ranges of 70–100, 80–90, and 90–100 to summarize pulse oximeter performance in various saturation groups. FDA guidelines for accuracy testing were used to measure at least 200 data points as paired SpO_2_−SaO_2_ observations balanced across each decadal range of SaO_2_ [17]. As previously mentioned, FDA guidance also recommends a sample size of at least 10 healthy subjects that vary in age and gender, with a range of skin pigmentation, including at least two darkly pigmented subjects or 15% of your subject pool, whichever is larger.

#### Low perfusion testing

In the low perfusion group, the multiple step desaturation method was used to collect the data pairs in SpO_2_ plateaus distributed evenly over the SpO_2_ accuracy range of 70–100%. For each subject, the stepwise desaturation process to achieve the 70% SpO_2_ level was repeated twice with a high SpO_2_ period and sensor rotation between. The target was to achieve ten SpO_2_ plateaus with each subject and in each SpO_2_ plateau to collect two test hand—control hand SpO_2_ data pairs.

Each subject’s left arm was submerged in an ice bath while the right arm was kept warm to serve as a control. In this group of healthy volunteers there were no expected baseline perfusion differences between the right and left arm, so the left arm was used in all subjects for consistency of experimental setup. Due to the time required to develop low perfusion in the experimental arm and the time that would have been required to recover that extremity to normal perfusion and immerse the opposite arm, rotation of the test and control arms was not feasible. The length of submersion was determined by the Perfusion Index (PI) as measured by the GE SpO_2_ device. PI is calculated as the ratio of pulsatile blood flow divided by the non-pulsatile blood flow times 100. Left arm cooling was performed until a PI value of less than 0.3% was reached, or a maximum of 60 min.

Pulse oximetry measurements were recorded continuously at each saturation plateau and SpO_2_ readings were compared between the test and control hands. PI values were recorded and grouped into five perfusion ranges: All, normal (PI ≥ 1.0), low (0.3 ≤ PI < 1.0), very low (0.1 ≤ PI < 0.3), and ultra low (PI < 0.1) to allow us to assess pulse oximeter performance across the various perfusion groups. The number of datapoints was equal across the subjects.

### Data analysis

**Fig. 1:**
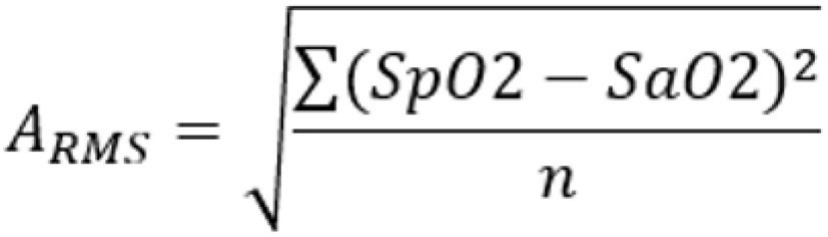
Formula for ARMS calculation

Statistical analysis was conducted with SAS 9.4. Descriptive data for comparison included the Accuracy Root Mean Square (ARMS) and bias. In the motion group, ARMS and bias was calculated as SpO_2_ minus the SaO_2_ reference value, with SaO_2_ serving as the reference. In the low perfusion group, the control SpO_2_ served as the reference. ARMS was calculated as the square root of the mean of the squared difference between test and reference values (Fig. 1).

ANOVA with post-hoc Dunnett test was used for comparison of mean biases. The homogeneity of ARMS was tested with Levene’s test. Bland–Altman method was used to visualize the relationship between tested SpO_2_ measurements against the reference method and to determine limits of agreement.

### Materials

All study devices (GE HealthCare CARESCAPE ONE TruSignal SpO_2_ Parameter, Masimo RADICAL-7, Medtronic Nellcor PM1000N SpO_2_) were CE marked and 510(k) cleared by the US FDA. Disposable adhesive sensors were used to prevent sensor displacement.

In the motion group, a 22-gauge radial arterial catheter was used for sampling reference co-oximetry oxyhemoglobin (SaO_2_) measurements on the control extremity. Blood gas analysis to determine SaO_2_ was performed with the ABL-90 multi-wavelength oximeter (Hemoximeter, Radiometer, Copenhagen, Serial 1393-090R0359N0002). In the low perfusion group, PI values were collected using the GE SpO_2_ device.

Ethically, to minimize the study risks for the subjects an arterial line was used only in the motion group. Arterial blood samples were collected to allow for comparison of device accuracy against the gold standard SaO_2_ in both non-motion and motion conditions. In the low perfusion group, the same secondary standard pulse oximeter device and model was used for SpO_2_ measurements on both the warm control hand and the cooled test hand, as an alternative to invasive testing, pulse oximetry standard ISO 80601-2-61:2017 annex EE.3 proposes a non-invasive comparison of SpO_2_ device accuracy through a validated secondary standard pulse oximeter. Calibration of the secondary oximeter is directly traceable to a CO-oximeter and thus serves as the transfer standard.

## Results

### Demographics

In the motion group (N = 14), 5 women and 9 men were included, with an age range of 24–43 years and a mean age of 28.1 (SD = 5.2) years. Skin tones varied by the Fitzpatrick scale as Type II (N = 1), Type III (N = 6), Type IV (N = 5), Type V (N = 1), and Type VI (N = 1). Ethnicity varied Asian (N = 5), Caucasian (N = 5), Hispanic (N = 2), Black (N = 1), and Multiethnic (N = 1).

In the low perfusion group (N = 14), 9 women and 5 men were included, with an age range of 20–48 and a mean age of 28.7 (SD = 7.8) years. Skin tones varied by the Fitzpatrick scale as Type II (N = 4), Type III (N = 5), Type IV (N = 3), Type V (N = 1), and Type VI (N = 1). Ethnicity varied Asian (N = 4), Caucasian (N = 6), African American (N = 1), and Multiethnic (N = 3).

### Non-motion results-control SpO_2_ vs. SaO_2_

**Fig. 2:**
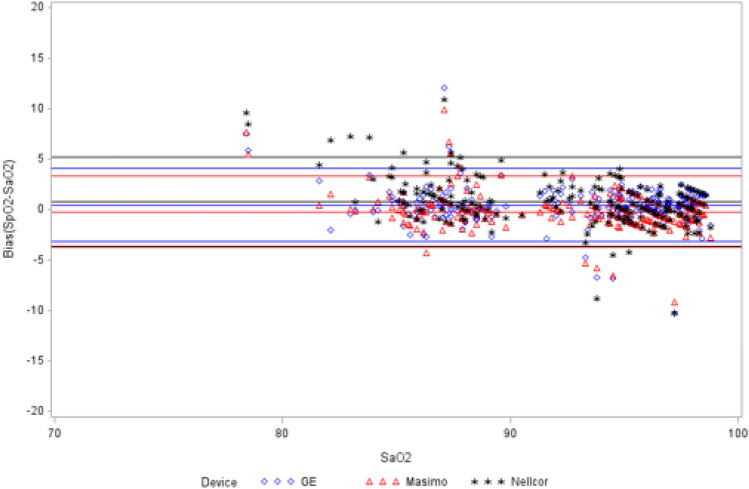
Bland–Altman plot for non-motion SaO_2_ range 70–100

The bias and ARMS results of the non-motion, control SpO_2_ sensors versus reference SaO_2_ are presented in Table 1. and Fig. 2. For the SaO_2_ range of 70–100, mean bias was less than 1 for each of the three devices, ARMS was lowest for Masimo at 1.79 and highest for Nellcor at 2.40. For SaO_2_ range of 80–90, mean bias was lowest for Masimo at 0.28 and highest for Nellcor at 1.68. ARMS was lowest for Masimo at 2.00 and highest for Nellcor at 2.89. For SaO_2_ range of 90–100, mean bias was less than 1 for each device, ARMS ranged from 1 to 2 for all devices. No significant differences in ARMS between devices were found in any of these comparisons. Mean bias measures were significantly different for each analysis range (P < 0.0001).

**Table 1:** Comparison of bias (SpO_2_–SaO_2_) and ARMS in non-motion hand

	Statistic	Masimo	Nellcor	GE	P-value
70-100	Mean	− 0.21^†^	0.78	0.45	<.0001
	Count	250	250	248	–
	Missing data	0	0	2	–
	Standard deviation	1.78	2.27	1.83	–
	Standard error	0.11	0.14	0.12	–
	95% CI	0.44 (− 0.44, 0.01)	0.57 (0.52, 1.09)	0.46 (0.23, 0.69)	–
	Limits of agreement	− 3.70 to 3.27	− 3.67 to 5.23	− 3.14 to 4.04	–
	Maximum	9.90	10.90	12.05	–
	Minimum	− 9.20	− 10.20	− 10.31	–
	Root mean square	1.79	2.40	1.88	0.1491
	Masimo vs GE (ARMS)				0.8538
	Nellcor vs GE (ARMS)				0.1366
	Nellcor vs Masimo (ARMS)				0.0722
80-90	Mean	0.28	1.68^†^	0.46	<.0001
	Count	82	82	81	–
	Missing data	0	0	1	–
	Standard deviation	2.00	2.37	2.04	–
	Standard error	0.22	0.26	0.23	–
	95% CI	0.88 (− 0.16, 0.72)	1.05 (1.16, 2.21)	0.91 (− 0.01, 0.90)	–
	Limits of agreement	− 3.63 to 4.19	− 2.97 to 6.32	− 3.54 to 4.45	–
	Maximum	9.90	10.90	12.05	–
	Minimum	− 4.30	− 2.20	− 2.68	–
	Root mean square	2.00	2.89	2.08	0.6780
	Masimo vs GE (ARMS)				0.9358
	Nellcor vs GE (ARMS)				0.4936
	Nellcor vs Masimo (ARMS)				0.3605
90-100	Mean	− 0.54^†^	0.24	0.37	<.0001
	Count	166	166	165	–
	Missing data	0	0	1	–
	Standard deviation	1.42	1.87	1.59	–
	Standard error	0.11	0.15	0.12	–
	95% CI	0.44 (− 0.76, − 0.32)	0.58 (− 0.03, 0.55)	0.49 (0.14, 0.64)	–
	Limits of agreement	− 3.33 to 2.24	− 3.43 to 3.90	− 2.75 to 3.50	–
	Maximum	3.30	4.10	3.02	–
	Minimum	− 9.20	− 10.20	− 10.31	–
	Root mean square	1.52	1.88	1.63	0.3711
	Masimo vs (ARMS)				0.9358
	Nellcor vs GE (ARMS)				0.4936
	Nellcor vs Masimo (ARMS)				0.3605

### Motion results-test conditions SpO_2_ vs. SaO_2_

**Fig. 3:**
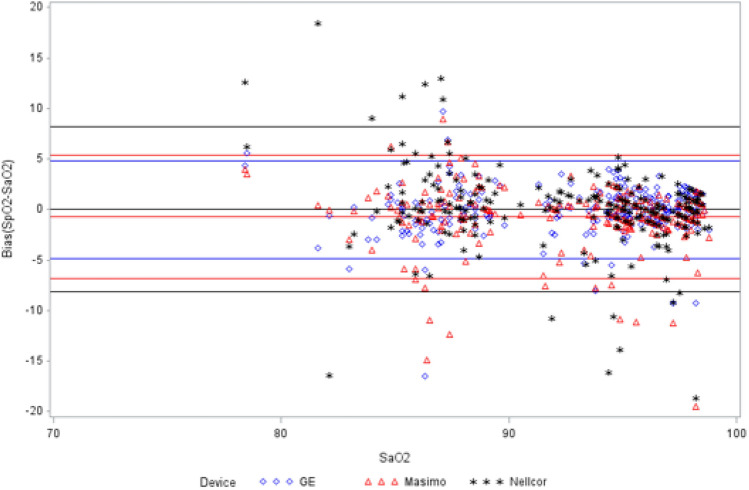
Bland–Altman plot for motion SaO_2_ range 70–100

The bias and ARMS results of the test SpO_2_ sensors during motion versus reference SaO_2_ are presented in Table 2 and Fig. 3. For the whole covered SaO_2_ range of 70–100, mean bias was less than 1 for each device, with ARMS lowest for GE at 2.45 and highest for Nellcor at 4.15. For SaO_2_ range of 80–90, mean bias was less than 1 for GE and Masimo and 1.07 for Nellcor. ARMS was lowest for GE at 3.01 and highest for Nellcor at 5.31. For SaO_2_ range of 90–100, mean bias was less than 1 for each device, ARMS was lowest for GE at 2.06 and highest for Nellcor at 3.29. Significant differences in bias were observed in all analyzed ranges (P < 0.05). Significant differences in ARMS were observed in the 70–100 range across all devices (P < 0.005) and between GE and Nellcor at each analyzed range (P < 0.05).

**Table 2:** Comparison of bias (SpO_2_–SaO_2_) and ARMS in motion hand

SaO_2_ Range	Statistic	Masimo	Nellcor	GE	P-value
70-100	Mean	− 0.75^†^	0.04	− 0.01	0.0121
	Count	250	250	248	–
	Missing data	0	0	2	–
	Standard deviation	3.10	4.16	2.45	–
	Standard error	0.20	0.26	0.16	–
	95% CI	0.77 (− 1.13, − 0.36)	1.04 (− 0.47, 0.56)	0.61 (− 0.29, 0.32)	–
	Limits of agreement	− 6.83 to 5.33	− 3.67 to 5.23	− 3.14 to 4.04	–
	Maximum	8.90	18.40	9.72	–
	Minimum	− 19.53	− 20.55	− 16.50	–
	Root mean square	3.19	4.15	2.45	0.0032
	Masimo vs GE (ARMS)				0.1292
	Nellcor vs GE (ARMS)				0.0020
	Nellcor vs Masimo (ARMS)				0.0513
80-90	Mean	− 0.38	1.07^†^	− 0.39	0.0338
	Count	82	82	81	–
	Missing data	0	0	1	–
	Standard deviation	3.73	5.23	3.00	–
	Standard error	0.41	0.58	0.33	–
	95% CI	1.64 (− 1.20, 0.44)	2.30 (− 0.08, 2.22)	1.28 (− 1.01, 0.27)	–
	Limits of agreement	− 7.70 to 6.93	− 2.97 to 6.32	− 3.54 to 4.45	–
	Maximum	8.90	18.40	9.72	–
	Minimum	− 14.90	− 20.55	− 16.50	–
	Root mean square	3.73	5.31	3.01	0.0520
	Masimo vs GE (ARMS)				0.3346
	Nellcor vs GE (ARMS)				0.0387
	Nellcor vs Masimo (ARMS)				0.1288
90-100	Mean	− 0.98	− 0.57	0.11^†^	0.0011
	Count	166	166	165	–
	Missing data	0	0	1	–
	Standard deviation	2.71	3.25	2.06	–
	Standard error	0.21	0.25	0.16	–
	95% CI	0.83 (− 1.40, − 0.57)	0.99 (− 1.07, −0.08)	0.64 (− 0.17, 0.47)	–
	Limits of agreement	− 6.29 to 4.33	− 3.43 to 3.90	− 2.75 to 3.50	–
	Maximum	3.30	5.20	3.94	–
	Minimum	− 19.53	− 18.70	− 9.28	–
	Root mean square	2.87	3.29	2.06	0.1327
	Masimo vs GE (ARMS)				0.3346
	Nellcor vs GE (ARMS)				0.0387
	Nellcor vs Masimo (ARMS)				0.1288

### Low perfusion results-test SpO_2_ vs. control SpO_2_

**Fig. 4:**
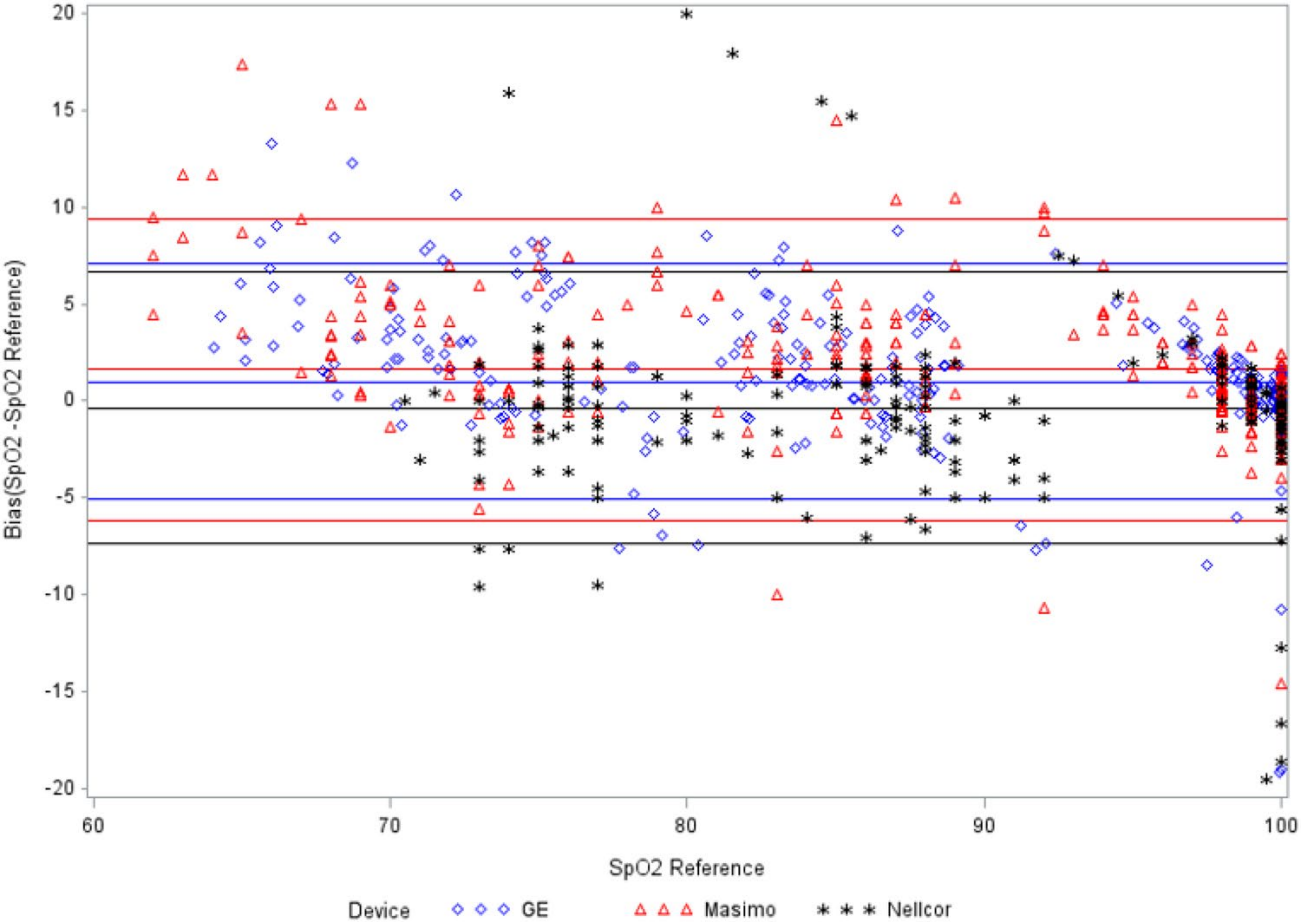
Bias (SpO_2_–SpO_2_ reference) for all perfusion ranges

The bias and ARMS results of the test SpO_2_ sensors versus reference SpO_2_ sensor are presented in Table 3. and Fig. 4. For all perfusion ranges, mean bias was the lowest for Nellcor at − 0.35 and greatest for Masimo at 1.62 (p < 0.0001). ARMS was lowest for GE at 3.26 and highest for Masimo at 4.30 (p = NS). For low PI, the mean bias was the lowest for Nellcor at − 0.53 and highest for Masimo at 1.76 (p < 0.0001). ARMS was the lowest for GE at 3.24 and highest for Masimo at 4.76 (p = NS). At very low PI, mean bias was the lowest for Nellcor at − 0.29 and highest for Masimo at 2.36, ARMS was lowest for GE at 4.52 and highest for Nellcor at 5.55 (p = NS for both mean and ARMS).

**Table 3:** Comparison of bias (SpO_2_–SpO_2_ Reference) and ARMS in low perfusion

PI Range	Statistic	Masimo	Nellcor	GE	P-value
Ultra low, PI < 0.1	Count	21	21	21	–
	Missing	9	10	0	–
	Mean	3.09	− 3.65^†^	0.47	0.0023
	Standard deviation (precision)	2.67	5.54	4.37	–
	Root mean square	4.01	6.42	4.29	0.4935
	Masimo vs GE (ARMS)				0.2182
	Nellcor vs GE (ARMS)				0.6016
	Nellcor vs Masimo (ARMS)				0.3371
Very low, 0.1 ≤ PI < 0.3	Count	51	33	51	–
	Missing	10	4	6	–
	Mean	2.36	− 0.29	1.65	0.0770
	Standard deviation (precision)	4.85	5.64	4.25	–
	Root mean square	5.33	5.55	4.52	0.6170
	Masimo vs GE (ARMS)				0.5475
	Nellcor vs GE (ARMS)				0.3577
	Nellcor vs Masimo (ARMS)				0.6322
Low, 0.3 ≤ PI < 1.0	Count	258	225	258	–
	Missing	21	3	2	–
	Mean	1.76	− 0.53^†^	0.96	<.0001
	Standard deviation (precision)	4.43	3.45	3.10	–
	Root Mean Square	4.76	3.48	3.24	0.1217
	Masimo vs GE (ARMS)				0.0678
	Nellcor vs GE (ARMS)				0.5537
	Nellcor vs Masimo (ARMS)				0.1954
Normal, PI ≥ 1.0	Count	93	84	93	–
	Missing	3	12	0	–
	Mean	0.71	0.69	0.91	0.6504
	Standard deviation (precision)	1.66	1.66	1.95	–
	Root mean square	1.80	1.78	2.14	0.5944
	Masimo vs GE (ARMS)				0.3912
	Nellcor vs GE (ARMS)				0.4629
	Nellcor vs Masimo (ARMS)				0.9786
All perfusion ranges	Count	423	363	423	–
	Missing	43	29	8	–
	Mean	1.62	− 0.35^†^	1.00	<.0001
	Standard deviation (precision)	3.99	3.56	3.11	–
	Root mean square	4.30	3.57	3.26	0.2175
	Masimo vs GE (ARMS)				0.0832
	Nellcor vs GE (ARMS)				0.3210
	Nellcor vs Masimo (ARMS)				0.4569

## Discussion

This study adds to the existing body of evidence on pulse oximeter performance under conditions of motion and low perfusion. We believe this is the first study to induce significant levels of low perfusion using an ice bath test method.

The non-motion test results showing the measurements of the control hand versus SaO_2_ shows minimal bias in all three devices and the ARMS showed no significant differences. In this scenario, the clinical challenges to SpO_2_ measurement accuracy are minimal, simulating pulse oximetry measurement in a patient with normal perfusion and little to no motion, such as during elective procedural care or outpatient medicine. This finding is consistent with previous research, which has found similar performance across most SpO_2_ devices [14, 15].

An ongoing concern is the potential impact of skin pigmentation levels on SpO_2_ measurements, which have been previously reported [9, 10, 21, 22]. While we included subjects with a range of skin pigmentation, this study was not designed to specifically assess the impact of skin pigmentation on SpO_2_ performance, and thus this remains an area for future study.

The finding of two oximeters (Nellcor and GE) having reduced bias during motion versus non-motion conditions for the SaO_2_ range of 70–100 was unexpected, since performance is normally decreased during motion. Because the differences were small, the observation may be explained by limitations in measurement accuracy. The mean bias of − 0.75 observed with Masimo during motion conditions, versus a mean bias of 0.78 with Nellcor during non-motion conditions, suggests that this degree of error may fall within the accuracy limits of SpO_2_ performance. More importantly, a bias less than 1 may not be clinically significant when interpreted with additional data describing the clinical condition of the patient.

A study by Louie et al., found the ARMS error greater than 3% in motion test conditions in all devices except for Nihon Kohden [13]. In the 70–100% saturation range used in this study, the GE device had an ARMS error of 2.45, while Nellcor and Masimo had ARMS of 4.15 and 3.19 respectively (P < 0.005). However, overall the SpO_2_ performance was similar among the three devices and is consistent with the findings reported by Louie.

The low perfusion test conditions resulted in greater performance degradation and larger mean bias and ARMS values. The normal PI of ≥ 1.0 is associated with mean bias levels less than one, but ARMS values ranged from 1.78 (Nellcor) to 2.14 (GE). In this study normal PI is defined as ≥ 1.0, while in a previous study PI values < 2 were considered representative of poor perfusion [13].

In the low and ultra low PI ranges, significant differences in mean bias were observed across devices. In the very low PI range, no significant differences were evident. At ultra low levels of perfusion, Masimo (N = 9) and Nellcor (N = 10) experienced a number of missing values while GE had no missing values. The increase in proportion of missing values with ultra low PI, suggests that a threshold for pulse oximeter performance may have been reached although additional study is required to evaluate further. However, due to the low number of low and ultra low PI samples, no conclusions can be reached regarding relative pulse oximeter performance. Additional studies using larger patient populations and data sets which include more low and ultra low PI samples are required compare performance across different SpO_2_ devices.

Loss of pulse oximetry signals due to low perfusion is a clinical challenge requiring additional actions to estimate arterial oxygen levels and hemoglobin saturation. If the signal is lost from a finger, probes may be applied on alternative anatomic locations such as toes, ears, buccal mucosa, or nares. Invasive measurement via arterial blood gas is a clinical option when the pulse oximetry signal is not reliable. However, invasive measurement is associated with increased risk of patient morbidity due to line placement, line dislodgement, repeated blood sampling, delays due to the requirement to transport and run samples, anemia if repeated measurement is required, and costs from supplies and equipment use [23–25].

The motion testing group assessed pulse oximetry performance over a wide range of clinically relevant conditions. Findings demonstrated similar performance of all three SpO_2_ devices even though some statistically significant differences in bias and ARMS were observed. The accuracy of SpO_2_ measurement during low perfusion conditions showed a greater degree of degradation when compared to normal perfusion, with statistically significant differences in bias found primarily in the low perfusion measurements. These study findings highlight the limitations of pulse oximetry technologies which are dependent on pulsatile blood for accurate measurement. Since conditions of low perfusion are common in the clinical setting, it is important to recognize that pulse oximeter measurements without the context of other relevant clinical data, are often not sufficient to guide diagnostic and therapeutic clinical decisions.

### Limitations

As this study was conducted in a controlled laboratory setting, it is unlikely that we were able to fully replicate or adequately represent device performance in the actual clinical environment. This study was conducted using healthy volunteer subjects without significant illness or comorbidity, which is not representative of a typical patient population, particularly in acute care. Moreover, our sample for subjects with darker skin pigmentation was small, limiting the use of these results in this patient population. The study was not powered to examine the impact of motion or low perfusion on any patient subgroups. Finally, because of the low number of observations of ultra low PI (N = 21), no meaningful conclusions of SpO_2_ comparative performance can be made at this PI strata.

## Conclusion

The overall finding from this study is that performance of all three SpO_2_ devices was similar across simulated motion and low perfusion conditions. Consistent with previous research on the impact of motion, the SpO_2_ measurement accuracy degraded for all three devices when compared to non-motion controls. For all three devices, accuracy also degraded as the perfusion index was reduced.

Pulse oximetry innovations to improve the quality, accuracy, and consistency of SpO_2_ measurements during clinical use are needed to improve patient safety. Continued technology development and additional studies are required to further improve SpO_2 _measurement accuracy and mitigate for limitations of use during motion, low perfusion, and in patients with darker skin pigmentation.
